# Impact of 2018 Japan floods on allergic rhinitis prescriptions^☆^

**DOI:** 10.1016/j.waojou.2025.101051

**Published:** 2025-04-23

**Authors:** Hanae Konishi, Hiroshi Iwamoto, Shuhei Yoshida, Yasushi Horimasu, Shinichiro Ohshimo, Kota Takemoto, Noboru Hattori, Sachio Takeno, Masatoshi Matsumoto

**Affiliations:** a Department of Molecular and Internal Medicine, Graduate School of Biomedical and Health Sciences, Hiroshima University, 1-2-3 Kasumi, Minami-ku, Hiroshima 734-8551, Japan; b Department of Community-Based Medical Systems, Graduate School of Biomedical and Health Sciences, Hiroshima University, 1-2-3 Kasumi, Minami-ku, Hiroshima 734-8551, Japan; c Department of Emergency and Critical Care Medicine, Graduate School of Biomedical and Health Sciences, Hiroshima University, 1-2-3 Kasumi, Minami-ku, Hiroshima 734-8551, Japan; d Department of Otorhinolaryngology, Head & Neck Surgery, Graduate School of Biomedical and Health Sciences, Hiroshima University, 1-2-3 Kasumi, Minami-ku, Hiroshima 734-8551, Japan

**Keywords:** Climate change, Epidemiology, Natural disaster, Rhinitis

## Abstract

**Background:**

Climate change and natural disasters can impact allergic conditions. The 2018 Japan floods, which occurred in July 2018, were among the largest water-related disasters in Japan's history. We aimed to investigate the impact of flooding on prescription rates for allergic rhinitis.

**Methods:**

This retrospective cohort study utilized data from the National Database of Health Insurance Claims from 1 year before and after the flood in the most severely affected region. Individuals with a victim code certified by local authorities were categorized into the victim group, whereas the others were classified into the non-victim group. A difference-in-differences analysis with a logistic regression model was employed to evaluate the impact of the disaster on prescription rates of corticosteroids or antihistamine nasal sprays. Cedar and cypress pollen (the major allergens causing seasonal rhinitis during spring in Japan) counts were measured using a rotary-type pollen collector.

**Results:**

Among 6,176,299 individuals included, 36,076 were identified as flood victims. An upward trend in prescriptions was observed during the cedar and cypress pollen season from February to April, and the pattern of higher prescriptions among disaster victims relative to non-victims continued throughout both the pollen and non-pollen seasons after the disaster. The difference-in-differences analysis indicated a significant increase in nasal spray prescription in disaster victims compared with non-victims, with adjusted odds ratios of 1.40 (95% confidence interval: 1.24–1.58) 3 months after the disaster and 1.72 (95% confidence interval: 1.56–1.95) 1 year after. Subgroup analyses showed that the prescription rates of nasal spray significantly increased across all age groups and in both males and females among disaster victims compared to non-victims.

**Conclusions:**

This study demonstrated a long-term increase in prescriptions for allergic rhinitis among flood victims, underscoring the need to recognize natural disasters as potential contributors to the incidence of allergic rhinitis.

## Introduction

Global warming is significantly contributing to an increase in heavy rainfall and disasters, possibly impacting respiratory health and allergic conditions.^1^, ^2^, ^3^, ^4^, ^5^, ^6^, ^7^ Allergic rhinitis is an allergic disease affecting the nasal mucosa, influenced by both allergens and environmental factors.^8^^,^^9^ Heavy rainfall and flooding can increase the risk of allergic diseases due to exposure to floodwaters, mold, pollutants, and psychological stress.^10^, ^11^, ^12^ However, no studies have established a link between heavy rain disasters and allergic rhinitis through population-based large-scale data.

From June 28 to July 8, 2018, torrential rains fell across western Japan, causing landslides and river flooding. The 2018 Japan floods were one of the most devastating events in Japan's disaster history, resulting in 263 deaths, 484 injuries, and 8 missing individuals. Additionally, the disaster impacted 29,473 houses, encompassing complete destruction, partial destruction, and flooding above floor level.^13^ The Japanese universal health insurance system ensures that all citizens are covered by public health insurance and can receive inexpensive treatment for allergic rhinitis with a doctor's prescription. In addition, the Japanese government database provides accurate and complete information on prescriptions for corticosteroid nasal sprays and antihistamine nasal sprays, as well as details on individuals certified as victims of the 2018 Japan floods by local governments. We proposed that analyzing large-scale longitudinal data on disaster victims and non-victims would allow us to determine the impact of flood disasters on allergic rhinitis.

This study aimed to determine whether the use of nasal sprays for allergic rhinitis increased due to 2018 Japan floods. We also conducted an exploratory analysis of data on cedar and cypress pollen counts, which are the primary allergens for seasonal allergic rhinitis in Japan.^8^

## Methods

### Study design and data collection

This retrospective cohort study utilized data from the National Database of Health Insurance Claims (NDB), operated by the Japanese government. The NDB records information on all prescription drugs dispensed in Japan and all medical insurance claims for all persons visiting a medical facility. The only exceptions are populations receiving public income support or those with automobile liability insurance or workers' compensation insurance, which account for less than 2% of the entire population. For this study, we used NDB data with permission from the Ministry of Health, Labour and Welfare. This study was conducted in accordance with the principles embodied in the Declaration of Helsinki and was approved by our university's Human Research Ethics Committee. The requirement for informed consent was waived owing to the anonymization of NDB data.

### Setting

From June 28 to July 8, 2018, western Japan experienced severe heavy rain disasters, leading to landslides and river flooding. The most severely affected areas were the Hiroshima, Okayama, and Ehime prefectures, which accounted for approximately 90% of the total number of fatalities, with estimated damages reaching approximately $12.66 billion. This disaster represents the largest damage scale recorded in Japan's history of heavy rain disasters. The observation period for this study was from July 2017 to June 2019, covering 1 year before and 1 year after the disaster. The study participants included individuals who visited medical institutions in the Hiroshima, Okayama, and Ehime prefectures for outpatient care or hospitalization during the observation period.

Data extracted from the NDB included information on sex, age groups (0–14 years, 15–59 years, and ≥60 years), names and quantities of prescribed medications, and the number of prescription days. During the 2018 Japan floods, victims certified by local governments and the Japanese government were fully exempted from the co-payment portion (10–30%) of their medical expenses. The victims were defined as: (1) those whose homes were completely destroyed, partially destroyed, burned down, flooded above floor level, or suffered similar damage, or (2) those who experienced death, injury, disappearance, unemployment, business cessation, or loss of income of family members who economically supported the disaster victims.

### Outcomes

The primary outcome was the proportion of monthly prescriptions for corticosteroids and antihistamine nasal sprays (Supplementary Table 1) covered by insurance in Japan for allergic rhinitis during the observation period. The secondary outcomes included stratified analysis by nasal spray type, age, and sex, as well as the proportion of patients prescribed second-generation oral antihistamines, which are considered the first-line treatment for allergic rhinitis. Further, we investigated whether pollen counts were associated with the prescription rate of nasal sprays for allergic rhinitis independent of background factors.

### Measurement of pollen counts

From January 10 to April 30 in 2017 and 2018, the degree of pollen dispersion was monitored by a gravitational pollen sampler on the roof of our university hospital. The cypress and cedar pollen counts were determined daily by staining with Calberla solution.^14^

### Data analysis

The chi-square test was used to compare dichotomous and categorical variables between victims and non-victims. First, we calculated the change in the percentage of the number of people prescribed nasal sprays and second-generation antihistamine oral drugs in victims and non-victims. Difference-in-differences (DID) analysis is a quasi-experimental method used to compare the changes in incidence of the target prescription over the observation period between victims and non-victims. The analysis theoretically excludes the influence of both unseen biases between victims and non-victims and biases arising over time.^15^^,^^16^ In this analysis, the degree of difference in prescription rates between the 2 groups remains constant in the absence of exposure (ie, the common trend assumption). The assumption is that any event would have affected the victims and non-victims equally (ie, the common shock assumption). In addition, we compared the occurrence of prescriptions of the target drug between May 2018 (2 months before the observed disaster) and each month after June 2018 for both victims and non-victims. Estimates of the change in the number of prescriptions for covered drugs among victims compared to those among non-victims were obtained from the ratio of odds ratios (ROR) by calculating them in a multivariate logistic regression model using an interaction term between time (each month after May 2015 versus May 2015) and victim status (victim versus non-victim). In each month after June 2018, 95% CIs calculated using the May 2018 value as the baseline adjusted ROR were reported. Thus, the ROR for each month indicates how many times the odds ratio increased compared with that for May 2018.

DID analysis was conducted using a linear probability model that included an interaction term with disaster status (victims vs. non-victims) when comparing the data from each month since June 2018 to that of May 2018. The analysis utilized robust standard errors for clusters (cluster subcommands) and considered repeated measurements by the same individual. The reference value was for May 2018, and estimates were reported using 95% CIs in each subsequent month.

Additionally, an exploratory multivariate logistic regression analysis was performed with nasal spray prescriptions as the objective variable and age, sex, disaster status, and pollen dispersal as explanatory variables in the 12 months before and 12 months after the disaster.

All statistical analyses were performed using STATA/MP-Parallel Edition version 16.1 (Stata Corp 2019). *P*-values <0.05 were considered statistically significant (two-tailed test).

## Results

### Participant demographics and pre-disaster nasal spray use

The study included 6,176,299 participants, consisting of 36,076 disaster victims and 6,140,223 non-victims. As shown in Table 1, compared to non-victims, the victims tended to include a higher proportion of older individuals and females. The proportion of individuals who received at least 1 prescription of nasal spray (antihistamine nasal spray and corticosteroid nasal spray) in the year before the disaster (July 2017 to June 2018) was the same for victims (7.3%) and non-victims (7.3%). Similarly, the proportions of individuals prescribed antihistamine nasal sprays and corticosteroid nasal sprays were 1.7% and 6.4% for disaster victims and 1.6% and 6.4% for non-victims, respectively, showing no significant difference.

**Table 1:** Baseline characteristics of study population before the disaster

	Victims	Non-victims	*P*-value
Number	36,076	6,140,223	
**Age category, no. (%)**			
0–14 years	3577 (9.9)	884,834 (14.4)	<0.001
15–59 years	11,649 (32.3)	2,761,247 (45.0)	
≥60 years	20,850 (57.8)	2,494,142 (40.6)	
**Sex, no. (%)**			
Male	16,006 (44.3)	2,905,192 (47.3)	<0.001
Female	20,070 (55.7)	3,235,031 (52.7)	
**Use of nasal spray prior to the disaster, no. (%)**			
Yes	2649 (7.3)	449,210 (7.3)	0.84
No	33,427 (92.7)	5,691,013 (92.7)	
**Use of antihistamine nasal spray prior to the disaster, no. (%)**			
Yes	599 (1.7)	97,800 (1.6)	0.31
No	35,477 (98.3)	6,042,423 (98.4)	
**Use of corticosteroid nasal spray prior to the disaster, no. (%)**			
Yes	2301 (6.4)	394,232 (6.4)	0.76
No	33,775 (93.6)	5,745,991 (93.6)	

### Trends in nasal spray prescriptions over time among disaster victims and non-victims

**Fig. 1:**
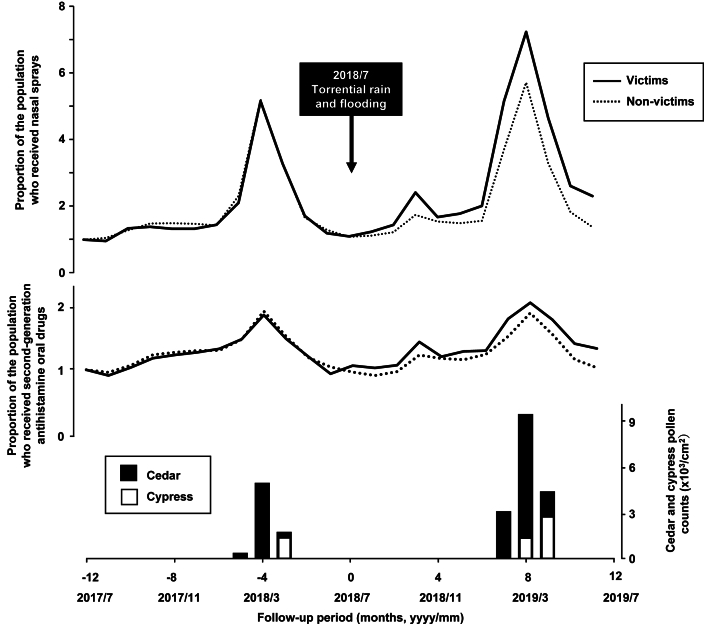
Amount of pollen and prescription rates of nasal sprays and second-generation antihistamine drugs before and after the disaster. The proportion of individuals prescribed nasal sprays and second-generation antihistamine oral drugs among victims and non-victims from July 2017 to June 2019 (top panel and middle panel, respectively). The proportions of prescriptions are plotted as ratios relative to the proportions of individuals prescribed nasal sprays in July 2017, with July 2017 proportions set at 1.0. The solid line represents victims, while the dashed line represents non-victims. The monthly transitions of pollen dispersion (bottom panel) are shown as closed bars for cedar pollen and open bars for cypress pollen. The horizontal axis denotes month 0 as July 2018, the month of the 2018 Japan floods

The proportions of individuals with prescriptions of nasal sprays and second-generation antihistamine oral drugs are plotted as ratios relative to the prescription numbers in July 2017 (Fig. 1). Since the July 2018 disaster, prescriptions for nasal sprays and antihistamines for allergic rhinitis have shown a higher trend among disaster victims compared to non-victims. The pollen counts in the 2019 pollen season were numerically higher than those in 2018. The monthly prescription numbers of nasal sprays and antihistamine oral drug are shown in Supplementary Tables 2 and 3 An upward trend in prescriptions was observed during the cedar and cypress pollen season from February to April, and the pattern of higher prescriptions among disaster victims relative to non-victims continued throughout both the pollen and non-pollen seasons (Fig. 1).

### Sustained one-year increase in nasal spray prescriptions among the victims after disaster

**Fig. 2:**
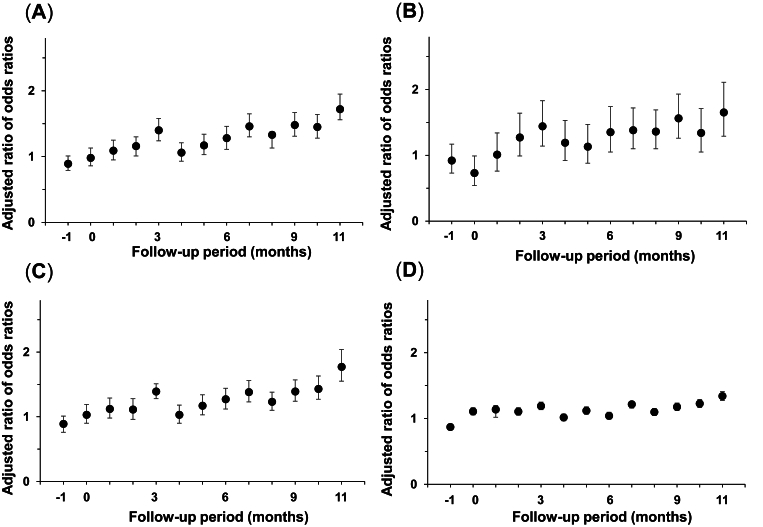
Prescriptions among victims versus non-victims evaluated using difference-in-differences analysis. Adjusted ratio of odds ratios for prescriptions of all nasal sprays (Fig. 2A), antihistamine nasal sprays (Fig. 2B), corticosteroid nasal sprays (Fig. 2C), and second-generation antihistamine oral drugs (Fig. 2D) between victims and non-victims. Month 0 is July 2018. The error bars indicate the 95% confidence intervals

Next, we present the results of DID analyses of differences in prescription rates between victims and non-victims (Fig. 2). The combined results of nasal sprays (antihistamine nasal sprays and corticosteroid nasal sprays) showed that the ROR for prescriptions increased 3 months after the disaster (adjusted ROR: 1.40; 95% CI: 1.24–1.58) and remained significantly increased 1 year after the disaster (adjusted ROR: 1.72; 95% CI: 1.56–1.95) (Fig. 2, A). The RORs for antihistamine and corticosteroid nasal sprays prescriptions were 1.44 (95% CI: 1.14–1.83) and 1.39 (95% CI: 1.21–1.58) 3 months after the disaster, and 1.65 (95% CI: 1.29–2.11) and 1.78 (95% CI: 1.55–2.04) 1 year after the disaster, respectively (Fig. 2B and C).

Additionally, we explored the effects of pollen counts on the prescription rate of nasal sprays for allergic rhinitis before and after the flood, after adjusting for background factors (Supplementary Table 4). Increased pollen counts and being a flood victim were independently associated with increased nasal spray prescription rates after the disaster.

### Higher ROR increase for nasal sprays compared to antihistamine oral drugs after the disaster

The ROR for the prescription of second-generation antihistamine oral drugs increased 3 months after the disaster (adjusted ROR: 1.18; 95% CI: 1.13–1.25) and remained significant 1 year after the disaster (adjusted ROR: 1.34; 95% CI: 1.27–1.41) (Fig. 2, D). The ROR for nasal spray prescriptions 12 months after the disaster was higher than that for second-generation antihistamine oral drugs, and the 95% CIs did not overlap.

### Nasal spray prescriptions increased across all age groups

**Fig. 3:**
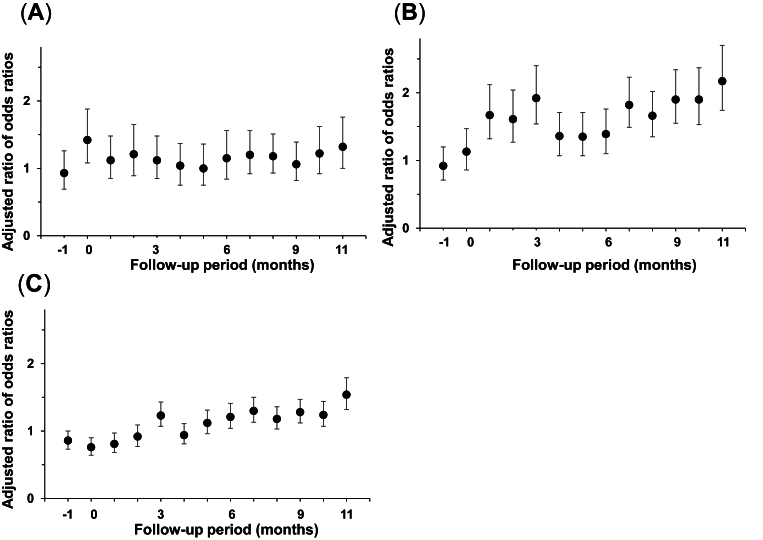
Prescriptions of all nasal sprays (including antihistamine nasal sprays and corticosteroid nasal sprays) among victims versus non-victims evaluated using difference-in-differences analysis, stratified by age group (A: 0–14 years; B: 15–59 years; C: ≥60 years). Month 0 is July 2018. The error bars indicate the 95% confidence intervals

**Fig. 4:**
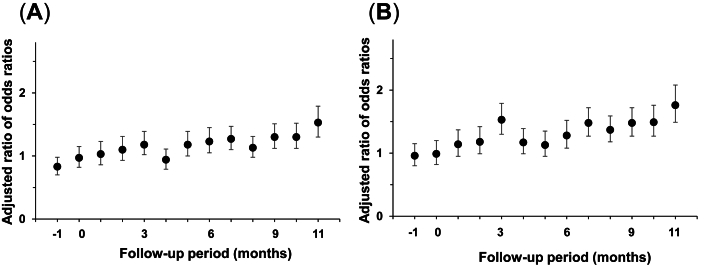
Prescriptions of all nasal sprays (including antihistamine nasal sprays and corticosteroid nasal sprays) among victims versus non-victims evaluated using difference-in-differences analysis, stratified by sex (A: males; B: females). Month 0 is July 2018. The error bars indicate the 95% confidence intervals

The ROR for nasal spray prescriptions by age group showed that the prescription rates were significantly increased in all age groups, with a trend toward a lower ROR in the 0–14 age group than in the other age groups (Fig. 3A–C). The ROR for nasal spray prescriptions by sex showed that the ROR increased in both males and females from 3 months after the disaster and remained high 1 year after the disaster (Fig. 4A and B). No obvious difference was found between the 2 groups.

## Discussion

This study examined whether the use of nasal sprays for allergic rhinitis increased following the floods by analyzing a large-scale longitudinal database while controlling for various confounders. We found an increased rate of nasal spray prescriptions for allergic rhinitis in victims compared with that in non-victims following the 2018 Japan floods. This increase became statistically significant 3 months after the flood and remained significant 12 months after. These results indicate that allergic rhinitis increases in victims of natural disasters, and this influence persists over the long term.

Previous studies reported a high prevalence of allergic rhinitis in areas affected by flood disasters.^12^^,^^17^^,^^18^ However, studies that only track patient groups after a disaster lack a control group, making it difficult to determine whether the worsening of patients' conditions is attributable to the disaster. Unlike in this study, the previous studies did not classify the residents of the affected areas into victims and non-victims and track them individually over a long period, limiting their ability to establish a causal relationship. Additionally, ecological analyses at the community level, which do not identify individuals, are prone to ecological fallacies, where results at the group level do not accurately reflect individual-level outcomes. In this study, we addressed the limitations of past research by identifying and comparing individuals within the same affected area who were directly impacted by the disaster with those who were not, thereby demonstrating the relationship between the disaster and the disease.

This study revealed that the increased prescription of intranasal sprays for allergic rhinitis among flood disaster victims persisted for 1 year. Oluyomi et al used a questionnaire survey among individuals who engaged in post-disaster cleanup and reported that sinus irritation was the most common allergic symptom 1 year after Hurricane Harvey, agreeing with our results.^12^ They also reported that exposure to mold, dirty water, and debris were risk factors of sinus irritation, and exposure to mold and flooded houses were risks for perceived stress 1 year after the disaster.^12^ Mold exposure can have both allergen and irritant effects, affecting epithelial barrier function, and playing a significant role in the pathogenesis of allergic rhinitis.^19^ Flooding and subsequent exposure to water-damaged dwellings increase the risk of mold exposure, which can persist for up to 1 year, as residents remain in contaminated homes until major remediation or reconstruction is completed.^20^, ^21^, ^22^ Exposure to environmental factors during the floods and post-disaster clean-up as well as prolonged psychological stress, as reported in the 2018 Japan floods,^23^ are factors that likely contributed to the development of allergic rhinitis. We further found that the number of nasal spray prescriptions was higher among individuals aged ≥15 than in those aged 0–14 years. This may be because adults and adolescents might have been more involved in disaster recovery efforts, which might have increased their exposure to allergens, pollutants, and stress, potentially contributing to a worsening of allergic rhinitis. In the present study, the adjusted ROR for nasal sprays at both 3 months and 1 year was higher (with non-overlapping 95% CIs) compared with that for second-generation antihistamine oral drugs. Second-generation antihistamine oral drugs are the first-line treatment for mild allergic rhinitis and other allergic conditions, whereas intranasal corticosteroid sprays are recommended for persistent or moderate-to-severe symptoms of allergic rhinitis.^2^ Intranasal corticosteroid spray users often have perennial and moderate-to-severe nasal symptoms. Therefore, our findings indicate that the floods may have led to a more severe form of allergic rhinitis and cedar pollinosis among the victims.

Our findings demonstrate that, in disaster victims, the demand for rhinitis treatment was increased both in the pollen season and in the non-pollen season compared with that in non-victims. Cedar and cypress pollen are the major allergens causing seasonal rhinitis from February to April in Japan.^8^ During this seasonal allergic rhinitis period, an overall increase in prescriptions was observed. The trend of higher prescription rates among disaster victims compared to non-victims persisted until 1 year after the disaster, including during the pollen season. The DID analysis demonstrated that the prescription of nasal sprays significantly increased among disaster victims compared to non-victims throughout both the pollen and non-pollen seasons following the disaster. Additionally, exploratory logistic regression analysis indicated that both disaster victim status and elevated pollen levels independently contributed to an increase in nasal spray prescriptions. Taken together, our findings indicate 2 points. First, the long-term effects of flood disasters, such as mold exposure, pollutants, and psychological stress, led to increased prescriptions for allergic rhinitis in both pollen and non-pollen seasons for 1 year. Second, in disaster victims, the seasonal allergic rhinitis period was associated with an additive increase in the demand for rhinitis treatment, primarily influenced by long-term effects from the disaster, with additional contributions from pollen allergen exposure.

This study has some limitations. First, because the co-payment was waived for victims, this could have increased the rate of nasal spray prescriptions. However, prices for prescribed drugs in Japan are relatively lower among developed countries, and the co-payment rate in the insurance system is no more than 30%, suggesting that affordability does not affect healthcare accessibility. The study population in the target area was 6.27 million (2018); our study covered over 90% of this population, indicating that most residents visited medical institutions at least once in the two-year period. This high coverage is due to the Japanese universal health insurance system, which provides easy access and low-cost co-payments. Additionally, the price elasticity (ie, the percentage change in quantity demanded when there is a 1%increase in price) of outpatient care in the Japanese health insurance system is reportedly very weak (−0.125 to −0.07619).^24^ The average co-payment rate for the disaster victim group in this study was as low as 22.3%, mainly due to the high proportion of elderly individuals in the target population. All these factors indicate that the influence of co-payment exemption was minimal. The second limitation is that over-the-counter medications were not included, possibly leading to an inadequate investigation of patients with mild nasal allergies and patients who are busy, middle-age working professionals. Third, the reasons for each patient's visit and diagnosis were not specified within the database. However, it is unlikely that nasal sprays would be prescribed for conditions other than rhinitis; thus, we believe that changes in prescription rates can sufficiently indicate the onset or exacerbation of allergic rhinitis among disaster victims and non-victims.

Considering the recent increase in heavy rain and floods due to global warming,^7^ the potential increase in allergic rhinitis during disasters has significant clinical and policy implications. For example, physicians who treat patients with allergic rhinitis should be aware of this phenomenon and anticipate an increase in patients with this condition during emergencies. This risk should be included in clinical guidelines. Additionally, policymakers should also anticipate an increased demand for these medications during emergencies and include them in disaster preparedness drug stockpiles, considering the global impacts of climate change.

In conclusion, this study demonstrated a sustained increase in prescription for allergic rhinitis among flood victims for 1 year after the disasters, indicating that natural disasters may contribute to a higher incidence of allergic rhinitis. These findings support the association between disasters and allergic disease and will aid in the post-disaster management of allergic rhinitis.

## Authors’ contributions

HK carried out the initial analyses, drafted the initial manuscript, and critically reviewed and revised the manuscript. SY carried out the initial analyses and critically reviewed and revised the manuscript. HI conceptualized and designed the study, drafted the initial manuscript, and critically reviewed and revised the manuscript. YH designed the study and critically reviewed and revised the manuscript. SO conceptualized and designed the study and critically reviewed and revised the manuscript. KT designed the study and critically reviewed and revised the manuscript. NH critically reviewed and revised the manuscript for important intellectual content. ST conceptualized and designed the study and critically reviewed and revised the manuscript. MM coordinated and supervised data collection; he critically reviewed and revised the manuscript for important intellectual content. All authors read and approved the final manuscript.

All authors have agreed to the publication of this manuscript.

## Ethics statement

This study was conducted in accordance with the principles embodied in the Declaration of Helsinki and was approved by the Epidemiology Research Ethics Committee of Hiroshima University (approval number: E-1688). The requirement for informed consent was waived owing to the anonymization of National Database of Health Insurance Claims data. For this study, we used NDB data with permission from the Ministry of Health, Labour and Welfare (permission number: 1223-2).

## Data availability statement

Raw data will not be shared due to restrictions stipulated by the Ministry of Health, Labour and Welfare.

## Funding

This research received no specific grant from any funding agency in the public, commercial, or not-for-profit sectors.

## Declaration of competing interest

ST received payment or honoraria for lectures, presentations, and speakers’ bureau participation from Sanofi, Tanabe Mitsubishi Pharmaceutical Co. The rest of the authors have no conflict of interest.
