# Supplementary material for: Impact of 2018 Japan floods on allergic rhinitis prescriptions
Source: World Allergy Organ J. 2025 Apr 23;18(5):101051. doi: 10.1016/j.waojou.2025.101051 (PMC12051157; doi:10.1016/j.waojou.2025.101051)
Supplement: Multimedia component 1 [file mmc1.docx]

**Supplementary Tables**

**Supplementary Table 1**

| **Category** | **Drug** |
| --- | --- |
| Nasal spray  　Antihistamine nasal spray | Sodium cromoglicate  Ketotifen fumarate  Levocabastine hydrochloride |
| Corticosteroid nasal spray | Beclomethasone dipropionate  Fulticasone propionate  Mometasone furoate hydrate  Fluticasone furoate  Dexamethasone cipecilate |
| Second-generation antihistamine drug | Ketotifen fumarate  Azelastine hydrochloride  Oxatomide  Mequitazine  Fexofenadine hydrochloride  Epinastine hydrochloride  Ebastine  Cetirizine hydrochloride  Levocetirizine hydrochloride  Bepotastine besilate  Emedastine fumarate  Olopatadine hydrochloride  Loratadine  Desloratadine  Bilastine  Rupatadine fumarate |

**Supplementary Table 2.** Number of nasal spray prescriptions after the disaster

| **Year/month** | **Non-victims (%)** | **Victims (%)** |
| --- | --- | --- |
| 2017/07 | 37,094 (0.60) | 239 (0.66) |
| 2017/08 | 39,020 (0.64) | 229 (0.63) |
| 2017/09 | 47,573 (0.77) | 321 (0.89) |
| 2017/10 | 55,189 (0.89) | 332 (0.92) |
| 2017/11 | 55,470 (0.90) | 317 (0.88) |
| 2017/12 | 54,894 (0.89) | 318 (0.87) |
| 2018/01 | 53,110 (0.86) | 346 (0.96) |
| 2018/02 | 86,113 (1.40) | 505 (1.40) |
| 2018/03 | 194,149 (3.16) | 1,241 (3.44) |
| 2018/04 | 121,939 (1.98) | 787 (2.18) |
| 2018/05 | 61,765 (1.01) | 410 (1.14) |
| 2018/06 | 48,050 (0.78) | 285 (0.79) |
| 2018/07 | 40,324 (0.66) | 263 (0.73) |
| 2018/08 | 41,517 (0.68) | 296 (0.82) |
| 2018/09 | 45,481 (0.74) | 345 (0.96) |
| 2018/10 | 64,797 (1.06) | 580 (1.61) |
| 2018/11 | 57,513 (0.94) | 402 (1.11) |
| 2018/12 | 55,572 (0.91) | 427 (1.18) |
| 2019/01 | 58,242 (0.95) | 483 (1.34) |
| 2019/02 | 138,021 (2.25) | 1,240 (3.44) |
| 2019/03 | 213,384 (3.48) | 1,741 (4.83) |
| 2019/04 | 122,682 (2.00) | 1,117 (3.10) |
| 2019/05 | 68,129 (1.11) | 627 (1.74) |
| 2019/06 | 51,196 (0.83) | 554 (1.54) |

**Supplementary Table 3.** Number of second-generation antihistamine drug prescriptions after the disaster

| **Year/month** | **Non-victims (%)** | **Victims (%)** |
| --- | --- | --- |
| 2017/07 | 347,162 (5.65) | 2,336 (6.48) |
| 2017/08 | 332,734 (5.42) | 2,126 (5.89) |
| 2017/09 | 370,734 (6.04) | 2,419 (6.71) |
| 2017/10 | 428,822 (6.98) | 2,760 (7.65) |
| 2017/11 | 441,803 (7.20) | 2,878 (7.98) |
| 2017/12 | 451,017 (7.35) | 2,975 (8.25) |
| 2018/01 | 452,322 (7.37) | 3,101 (8.60) |
| 2018/02 | 512,202 (8.34) | 3,454 (9.57) |
| 2018/03 | 665,101 (10.83) | 4,341 (12.03) |
| 2018/04 | 528,697 (8.61) | 3,469 (9.62) |
| 2018/05 | 414,019 (6.74) | 2,820 (7.82) |
| 2018/06 | 362,247 (5.90) | 2,187 (6.06) |
| 2018/07 | 334,392 (5.45) | 2,485 (6.89) |
| 2018/08 | 316,028 (5.15) | 2,405 (6.67) |
| 2018/09 | 337,079 (5.49) | 2,501 (6.93) |
| 2018/10 | 427,144 (6.96) | 3,354 (9.30) |
| 2018/11 | 408,171 (6.65) | 2,814 (7.80) |
| 2018/12 | 402,395 (6.55) | 3,008 (8.34) |
| 2019/01 | 430,858 (7.02) | 3,033 (8.41) |
| 2019/02 | 528,815 (8.61) | 4199 (11.64) |
| 2019/03 | 655,634 (10.68) | 4,788 (13.27) |
| 2019/04 | 538,414 (8.77) | 4,164 (11.54) |
| 2019/05 | 403,856 (6.58) | 3,290 (9.12) |
| 2019/06 | 357,865 (5.83) | 3,112 (8.63) |

**Supplementary Table 4.** Multivariate logistic regression models of nasal spray prescriptions before and after the disaster

| **Variables** | **Odds ratio (95% CI)** | ***P*-value** |
| --- | --- | --- |
| **Before the disaster (July 2017 to June 2018)** |  |  |
| Pollen dispersion (per ×10^3^/cm^2^ increase) | 1.203 (1.202–1.204) | <.001 |
| **After the disaster (July 2018 to June 2019)** |  |  |
| Pollen dispersion (per ×10^3^/cm^2^ increase) | 1.196 (1.195–1.197) | <.001 |
| Victims (versus non-victims) | 1.202 (1.159–1.248) | <.001 |

Sex and age groups were also included as independent variables in the model.
